# Postnatal exposure to PCB 153 and PCB 180, but not to PCB 52, produces changes in activity level and stimulus control in outbred male Wistar Kyoto rats

**DOI:** 10.1186/1744-9081-7-18

**Published:** 2011-05-26

**Authors:** Espen Borgå Johansen, Monica Knoff, Frode Fonnum, Per Leines Lausund, S Ivar Walaas, Grete Wøien, Terje Sagvolden

**Affiliations:** 1 Department of Physiology, Institute of Basic Medical Sciences, University of Oslo, Oslo, Norway; 2 Department of Biochemistry, Institute of Basic Medical Sciences, University of Oslo, Oslo, Norway; 3 Akershus University College, Kjeller, Norway; 4 Institute of Psychology, University of Oslo, Oslo, Norway; 5 The Norwegian Defense Research Establishment, Kjeller, Norway

## Abstract

**Background:**

Polychlorinated biphenyls (PCBs) are a class of organic compounds that bioaccumulate due to their chemical stability and lipophilic properties. Humans are prenatally exposed via trans-placental transfer, through breast milk as infants, and through fish, seafood and fatty foods as adolescents and adults. Exposure has several reported effects ranging from developmental abnormalities to cognitive and motor deficiencies. In the present study, three experimental groups of rats were orally exposed to PCBs typically found in human breast milk and then behaviorally tested for changes in measures of stimulus control (percentage lever-presses on the reinforcer-producing lever), activity level (responses with IRTs > 0.67 s), and responses with short IRTs (< 0.67 s).

**Methods:**

Male offspring from Wistar Kyoto (WKY/NTac) dams purchased pregnant from Taconic Farms (Germantown, NY) were orally given PCB at around postnatal day 8, 14, and 20 at a dose of 10 mg/kg body weight at each exposure. Three experimental groups were exposed either to PCB 52, PCB 153, or PCB 180. A fourth group fed corn oil only served as controls. From postnatal day 25, for 33 days, the animals were tested for behavioral changes using an operant procedure.

**Results:**

PCB exposure did not produce behavioral changes during training when responding was frequently reinforced using a variable interval 3 s schedule. When correct responses were reinforced on a variable interval 180 s schedule, animals exposed to PCB 153 or PCB 180 were less active than controls and animals exposed to PCB 52. Stimulus control was better in animals exposed to PCB 180 than in controls and in the PCB 52 group. Also, the PCB 153 and PCB 180 groups had fewer responses with short IRTs than the PCB 52 group. No effects of exposure to PCB 52 were found when compared to controls.

**Conclusions:**

Exposure to PCBs 153 and 180 produced hypoactivity that continued at least five weeks after the last exposure. No effects of exposure to PCB 52 were observed.

## Background

Polychlorinated biphenyls (PCBs) are a class of organic compounds that due to their chemical stability and resistance to degradation were used in a variety of products, including coolants and fluid oils for electric equipment (transformers, capacitors), plastics, and paints [1]. Although the manufacturing of PCBs was forbidden in the United States and Western Europe from around 1980, PCBs are still present in the environment due to their chemical stability and resistance to degradation, and accumulate in the food chain due to the lipophilic properties. Hence, humans are prenatally exposed via trans-placental transfer, through breast milk during infancy, and during adolescence and adulthood through consumption of contaminated food of which fish and seafood constitute the most important sources of PCB [1-3].

PCBs consist of two phenyl rings where chlorine can be substituted for hydrogen atoms, theoretically giving a total of 209 different PCB structures (congeners). The site and number of chlorine substitution(s) determine the molecule's chemical and toxic properties [4,5]. The congeners most commonly found in humans are the ortho-substituted non-planar PCBs 118, 138, 153, and 180, but also the less chlorinated di-ortho-substituted non-planar congener PCB 52 has been found [6-8]. Evidence suggests that even low-level exposure to PCBs during development can seriously impact neurobiological, cognitive, and behavioral functioning in humans and animals [2,9,10]. Prenatal exposure can produce abnormal pigmentation and several developmental abnormalities at birth including gum enlargement, presence of teeth, abnormal calcification of the scull, and low birth weight [11-13]. Neuroendocrine changes have been observed following prenatal exposure which can also interfere with neurotransmitter systems and signal transduction pathways [14] (for reviews, see [1,2]). PCB exposure affects neurological as well as cognitive and motor functions in humans [15-19]. Exposed humans show increased impulsivity, reduced attention and concentration, poorer working memory and lower IQ scores [8,18,20-24].

Controlled studies in animals indicate that several factors influence the measured effects of PCB exposure. These factors include dose, age when exposed and route of exposure, age when effects are tested and measures used, and sex and species tested [25-38]. Generally, however, findings from animal studies are consistent with research on humans, and show that PCB exposure affects learning and memory, activity level, and cognitive functions (for reviews, see [2,3]). The behavioral changes following exposure have been linked to alterations in monoamine function (dopamine and serotonin), vesicular transport and storage of monoamines, disruption of Ca^2+ ^signal transduction, and reduced long-term potentiation (LTP) (for reviews, see [1,2]).

The purpose of the present study was to examine behavioral changes in stimulus control (i.e. the percentage of responses on the reinforcer-producing lever), lever-directed activity level (responses with inter-response times, IRTs, > 0.67 s), and responses with short IRTs (< 0.67 s) in animals postnatally exposed to one of the three PCBs found in breast milk in humans (PCB 52, PCB 153, and PCB 180) compared to non-exposed controls. Outbred male Wistar Kyoto rats were orally given a mixture of corn oil and PCB at around postnatal day 8, 14, and 20 at a dose of 10 mg/kg body weight (bw) at each exposure. Thereafter, the animals were tested in standard operant test chambers using a simultaneous visual discrimination procedure developed for testing behavioral changes in an animal model of Attention-Deficit/Hyperactivity Disorder (ADHD) [39-41].

## Method

### Subjects

Male offspring from Wistar Kyoto (WKY/NTac) dams purchased pregnant from Taconic Farms (Germantown, NY) were used. During the first three weeks, the rats were under the care of a veterinarian at the Norwegian Defense Research Establishment, Kjeller, who also administered the PCBs. The mother animals were caged singly under standard laboratory animal conditions (temperature ~22°C, humidity ~55%, 12 hr light/dark cycle) in type IV macrolon cages and aspen bedding, where they also gave birth. Female offspring were culled at birth. The rats had free access to food (RM3 (E) from Special Diet Services, Witham, Essex CM8 3AD, UK) in the home cages at all times, and free access to water at all times.

At postnatal day (PND) 24, the rats were transported to the University of Oslo for behavioral testing. The rats were experimentally naïve on arrival. A total of 29 rats were behaviorally tested and included in the statistical analyses: The PCB 52 and PCB 180 groups each consisted of 9 animals, the PCB 153 group consisted of 6 animals, and the control group consisted of 5 animals.

During habituation and response acquisition, the rats were housed together in twos or threes in 41 × 25 × 25 (height) cm transparent cages. Following acquisition of lever-pressing and throughout the rest of the study, the rats were housed individually in the same type of cages. The rats had free access to food (RM3 (E) from Special Diet Services, Witham, Essex CM8 3AD, UK) in the home cage at all times, and free access to water at all times prior to the dipper training sessions. Starting with the dipper training session and throughout the rest of the study, the rats were deprived of water for 21 hours a day.

The temperature in the housing area was ~22°C, and the light was on from 0600 to 1800 hours. The behavioral training took place between 0900 and 1400 hours seven days a week, and lasted for 33 days.

The study was approved by the Norwegian Animal Research Authority (NARA), and was conducted in accordance with the laws and regulations controlling experiments/procedures in live animals in Norway.

### Apparatus

In the initial part of the study, sixteen Campden Instruments operant chambers were used. The chambers were located in two separate rooms each containing eight chambers and a separate computer. The number of operant chambers used was later reduced to eight due to a computer malfunction in one of the rooms (below). Each chamber was enclosed in a sound-resistant outer housing, was ventilated, and equipped with a grid floor. The animal's working space in eight of the chambers was 25 × 25 × 30 (height) cm (room 1), and 25 × 25 × 20 (height) cm in the other eight chambers (room 2). A fan producing a low masking noise and a 2.8-W house light were on during the entire experimental session. Each chamber was equipped with two retractable levers requiring a dead weight of at least 3 g to activate a micro-switch, and with a 2.8-W cue light located above each lever.

The reinforcers (0.05 ml tap water) were delivered by a liquid dipper located in a small recessed cubicle where a 2.8-W cue light lit up when a reinforcer was presented. A 7 × 5 cm transparent plastic top-hinged flap separated the cubicle from the animal's working space.

A computer program LabVIEW 7.1 recorded the behavior and scheduled reinforcers and lights [42].

### Procedure

*PCB exposure*. The animals were randomly assigned to one of the three experimental groups or to the control group and then orally given one of the three PCBs dissolved in corn oil or corn oil: Group 1 was fed corn oil only and served as controls; group 2 was fed PCB 52 (2,2',5,5'-Tetrachlorobiphenyl); group 3 was given PCB 153 (2,2',4,4',5,5'-Hexachlorobiphenyl); and group 4 was fed PCB 180 (2,2',3,4,4',5,5'-Heptachlorobiphenyl). The animals were exposed to PCB at around PND 8, PND 14, and PND 20 at a dose of 10 mg/kg body weight at each exposure. The total volume given at each exposure was 0.01ml/g body weight. Exposure was performed by the veterinarian during working hours, and was administered by gavage with stomach tube.

The PCBs, specially purified and free from dioxin-like PCBs, were purchased from Patrick Anderson, Department of Chemistry, University of Umeå, Sweden.

#### Habituation, dipper training, and response acquisition

Prior to behavioral testing, the rats were assigned an operant chamber and a time of testing in a semi-randomized and balanced way. Habituation to the operant chambers started at the day following arrival (PND 25) and lasted 30 min. During the habituation session, the flap between the working space and the reinforcement cubicle was taped open. No levers were present, the cue lights above the levers were off, and no reinforcers were delivered.

The habituation session was followed by two 30-min dipper training sessions. The flap was taped open, no levers were present, and the cue lights above the levers were off. The computer delivered water every 10 s independent of the animal's behavior using a fixed-time schedule. The cue light in the small recessed cubicle was turned on during each water delivery, and the reinforcer was available for 3 seconds.

In the following two sessions, the animals were trained to open the flap to gain access to the drop of water. The tape was removed from the flap, no levers were present, and the cue lights located above the levers were off. Each flap-opening turned on the cue light in the water cubicle and produced the presentation of a single drop of water. The water-dipper was lowered after 5 s irrespective of the animal's behavior.

During the subsequent two sessions, lever-pressing was shaped according to the method of successive approximations [43]. During the first of these sessions, the animals learned to press the left lever in order to receive a reinforcer immediately following every press. The cue light above the left lever was lit for the entire session except during presentation of the reinforcer when the light in the water cubicle was turned on. The right lever was retracted into the wall and the light above the lever was off. On the second session, the right lever was inserted and the left lever was retracted. During this session, the light above the right lever was lit the entire session except during presentation of the reinforcer when the light in the water cubicle was turned on. Immediately following response shaping on each lever, the animal was monitored to make sure the response was learned, and then left in the chamber for an additional 15 min to further strengthen the newly learned behavior. During this time, every press on the lever produced a reinforcer.

#### The variable interval 3 s schedule

Response acquisition was followed by five 30-min long training sessions (sessions 8-12) using a variable interval (VI) 3 s reinforcement schedule.

During the VI 3 s sessions and throughout the rest of the study, both levers were present. At the start of the session and following each reinforcer delivery, the computer program semi-randomly selected which lever produced the reinforcer. Lever selection was limited to a maximum of 4 consecutive reinforcers on the same lever to avoid the development of a lever-preference.

The lever producing the reinforcer was signaled (discriminative stimulus) by the lit cue light located above the lever. The light stayed lit for as long as the lever was associated with reinforcement, but was turned off during reinforcer presentation. The timer for the next interval started when the dipper was presented. Scheduled reinforcers and reinforcers produced, but not collected, were accumulated and scheduled for the next correct response.

Except for during the habituation and dipper training sessions, reinforcers were accessible for 3 s after the flap into the water cubicle was opened. Then, the dipper was lowered and the cubicle light was turned off. If the flap was not opened within 5 s after a reinforcer presentation, the water dipper was lowered and the cubicle light was turned off.

A concurrent extinction schedule was in effect on the alternative lever. The light above the alternative lever was always off. Thus, the present task can be described as a simultaneous visual discrimination task.

#### The VI 180 s schedule

A variable interval 180 s schedule (VI 180 s) was in effect for 90 min on one of the two levers from session 13 to session 19 when all sixteen operant chambers were used (Table 1). Due to a computer failure, all animals were tested in room 1 using 8 operant chambers from session 20 and throughout the remaining sessions. Also, session length was halved to 45 min to make it possible to test all animals during the day-time (see Table 1 for a summary of the experimental procedure). The malfunctioning computer scheduled reinforcers and lights correctly, but stopped on occasion without saving the data. The error did not differentially affect the groups as the groups were balanced across both test chambers and rooms.

**Table 1:** Summary of the experimental procedure

Session number	Schedule	Notes
1		Habituation

2 - 3	FT 10 s	Magazine training

4 - 5	CRF	Flap training

6 - 7		Shaping of lever-pressing

8 - 12^1^	VI 3 s	30 min session

13 - 19	VI 180 s	90 min session, 16 chambers

20 - 33^1^	VI 180 s	45 min session, 8 chambers

FT: fixed time schedule of reinforcement. CRF: continuous reinforcement schedule. VI: variable interval schedule of reinforcement.

Note. - ^1 ^Used in the analyses

A computer program was used to generate a Catania-Reynolds distribution of intervals for the VI 180 s schedule [44,45]. Inter-reinforcer intervals during the VI 180 s schedule ranged from 6 s to 719 s and were distributed in a semi-randomized fashion across the session. There was neither any external stimulus signaling that a reinforcer was programmed nor any external stimulus signaling the time since the last response.

#### Behavioral measures

The computer recorded number of presses on the lever producing reinforcers and on the alternative lever, number of flap openings to the cubicle, number of reinforcers produced and collected, and the time of the events. The following measures were calculated from the recorded behavior: Percentage of responses on the lever producing reinforcers, responses with IRTs longer than 0.67 s, and responses with IRTs shorter than 0.67 s.

In previous studies using the same operant procedure, the percentage of responses on the lever producing reinforcers was used as a measure of sustained attention (the animal has to pay attention to and press the lever signaled by the lit cue light located above the lever) [46-48]. Percentage correct would be at chance level (~50%) in animals pressing the reinforcement lever and the alternative lever equally often. Total number of lever-presses on the two levers combined was used as a measure of hyperactivity, and number of responses with short IRTs (< 0.67 s) was used as a measure of impulsivity ("premature responding" or "inability to wait"). Here, a neutral description of the measures will be used. The measures will be referred to as stimulus control, activity (level), and responses with short IRTs, respectively. Also, the number of responses with IRTs shorter than 0.67 s was subtracted from the measure of activity to ensure independency of the two measures.

### Data Analysis

All statistical analyses were done in Statistica 6.0 [49]. Data were evaluated by multivariate analyses using Wilks lambda (MANOVAs) when the degrees of freedom relative to the number of levels of the repeated factor permitted this approach, or by univariate analyses of variance (ANOVAs) adjusting the degrees of freedom with the Huynh-Feldt epsilon [50]. Sessions were used as the within-subject factor and treatment as the between-subjects factor. Post-hoc tests on main effects were performed using the Tukey HSD test.

**Figure 1:**
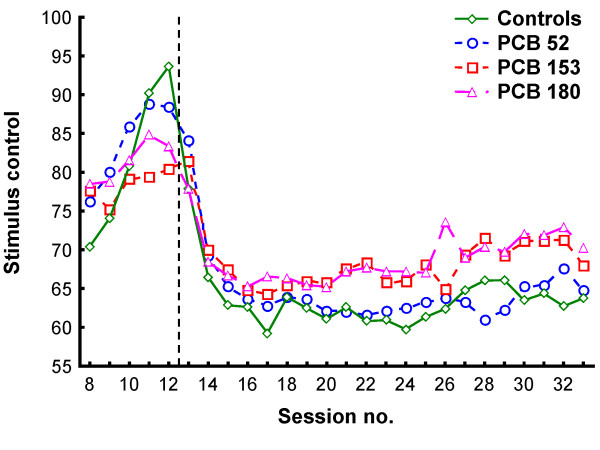
**Stimulus control (percentage of responses on the reinforcer-producing lever) in the four groups**. The vertical dotted line indicates when the contingencies changed from VI 3 s (sessions 8-12) to VI 180 s (sessions 13-33).

**Figure 2:**
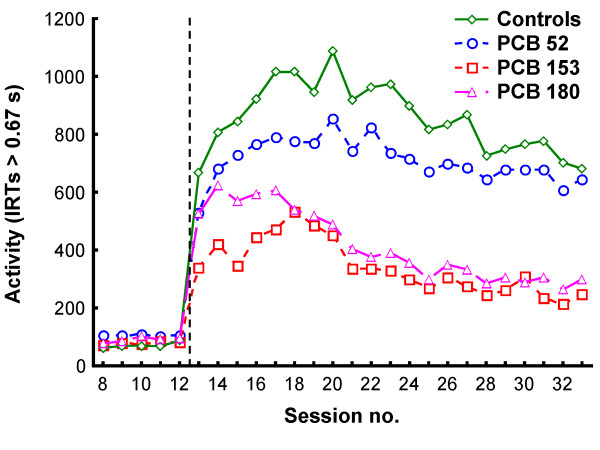
**The total number of lever-presses with IRTs > 0.67 s in the four groups**. The vertical dotted line indicates when the contingencies changed from VI 3 s (sessions 8-12) to VI 180 s (sessions 13-33).

**Figure 3:**
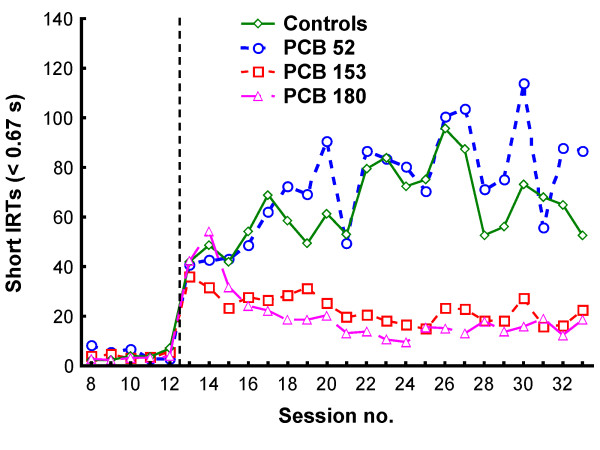
**The total number of lever-presses with short IRTs (< 0.67 s) in the four groups**. The vertical dotted line indicates when the contingencies changed from VI 3 s (sessions 8-12) to VI 180 s (sessions 13-33).

The five training sessions (sessions 8-12) under the VI 3 s schedule were analyzed separately. In a second analysis, the last 14 sessions (sessions 20-33) under the VI 180 s schedule were selected because these sessions represented relatively stable behavior. The cumulated numbers of responses at the end of each session were used in all analyses. In the figures showing responding across the sessions, data from the first 45 min were used in sessions 13 to 19 to match the 45-min session length in sessions 20 to 33 (Figures 1, 2 and 3).

One animal was able to collect reinforcers without activating the microswitch registering flap openings causing the computer to schedule too many reinforcers. As the reinforcement contingencies were unique for this animal, it was excluded from the statistical analyses.

Missing data were substituted by calculating the means of the preceding and following sessions. The Grubbs' test was used to identify and remove outliers [51]. A z-score exceeding the critical value represents a less than 5% probability of finding this value by chance alone. The critical value depends on number of subjects (N) and must be adjusted accordingly. Here, z-scores were calculated for each group of N = 5, N = 6, and N = 9 (two groups), and the critical values used for removing outliers in each group were, 1.71, 1.89, and 2.21, respectively. Examination of outliers identified by the Grubb's test for stimulus control, activity level (IRTs > 0.67 s), and number of responses with short IRTs (< 0.67 s) under the VI 180 s schedule showed that a total of 15 data points with an average z-score of 2.46 were removed for one animal. The remaining outliers identified and removed by the Grubb's test represented 0.9% of the total data in the VI 180 s condition.

## Results

Generally, stimulus control dropped from 80% during the VI 3 s schedule to between 60% and 70% during the VI 180 s schedule (Figure 1). Fewer lever-presses were observed under the VI 3 s schedule than under VI 180s due to the shorter session length and the larger proportion of time spent consuming the water (Figures 2 and 3).

### The VI 3 s schedule (sessions 8-12)

There were no statistically significant group differences in stimulus control, activity level (IRTs > 0.67 s), number of responses with short IRTs (< 0.67 s), number of flap openings, reinforcers produced, or reinforcers collected during training when responding was reinforced according to a VI 3 s schedule of reinforcement (Figures 1, 2 and 3). The analyses of activity level, however, showed a non-significant trend for a main effect of exposure, *F(3,22) = 2.85; p = 0.061*, with the PCB 52 group tending to be more active than the control, PCB 153 and PCB 180 groups (Figure 2; sessions 8-12).

### The VI 180 s schedule (sessions 20-33)

#### Stimulus control

The ANOVA showed a statistically significant main effect of exposure, *F(3,20) = 4.770; p = 0.011 *(Figure 1). Percentage of responses on the lever producing reinforcers increased across sessions, reflected in a statistically significant main effect of session, *F(13,260) = 2.161; p = 0.012*. No other effects were found. Tukey HSD post-hoc analyses of the significant main effect of exposure showed that this effect was produced by statistically significantly better stimulus control in the PCB 180 group compared to the control group and the PCB 52 group (*p*s *= 0.044*).

#### Activity (IRTs > 0.67 s)

The analysis showed a statistically significant main effect of exposure, *F(3,23) = 9.11; p < 0.001*. A statistically significant main effect of session showed that activity level decreased across sessions, *F(13,299) = 19.84; p < 0.001*. No interaction effect was found. Post-hoc analyses of the main effect of exposure using Tukey HSD tests showed that the PCB 153 and the PCB 180 groups were less active than the control group (*p = 0.010 *and *p = 0.004*, respectively) and the PCB 52 group (*p = 0.013 *and *p = 0.004*, respectively) (Figure 2).

#### Responses with short IRTs (< 0.67 s)

The ANOVA showed a statistically significant main effect of exposure, *F(3,20) = 5.06; p = 0.009*. No other effects were found. Tukey HSD post-hoc analyses of the main effect of exposure showed that the PCB 52 group had more responses with short IRTs than the PCB 153 and the PCB 180 groups (*p = 0.035 *and *p = 0.022*, respectively) (Figure 3).

#### Flap openings

There were no group differences in the number of visits to the water cubicle.

#### Reinforcers produced and reinforcers collected

There were no group differences in number of reinforcers produced. The analyses of number of reinforcers collected showed a trend for a main effect of exposure, *F(3,25) = 3.0; p = 0.051*. The average number of reinforcers collected was 14.06 in the control group and 13.82 in the PCB 153 group, with intermediate values in the two other groups.

To test that differences in number of reinforcers collected between experimental and control groups did not affect the statistical results, activity level (IRTs > 0.67 s) and number of responses with short IRTs (< 0.67 s) were divided by the number of reinforcers collected per session for each individual animal, and the data reanalyzed. The results showed the same statistically significant effects as when not correcting for reinforcers collected, with minimal changes in *p*-values.

## Discussion

The present study examined behavioral effects of exposure to PCB 52, PCB 153, and PCB 180 in outbred male Wistar Kyoto (WKY/NTac) rats. The PCBs were administered orally three times between postnatal day 8 and 20 at a dose of 10 mg/kg body weight at each exposure. Effects of exposure to the three ortho-substituted PCBs found in human breast milk were evaluated using a procedure developed to study behavioral changes in a rat model of Attention-Deficit/Hyperactivity Disorder [40,46]. The procedure uses an operant visual discrimination task in which the reinforced (signaled) lever switched randomly following every reinforcer-delivery to assess stimulus control (the discriminative control of a cue light on lever-presses, measured as percentage of responding on the lever producing reinforcers), activity level (total number of lever-presses with IRTs > 0.67 s), and responses with IRTs shorter than 0.67 s.

Data from two conditions were analyzed: During training when a VI 3 s schedule was in effect, and during a VI 180 s schedule when responses on the signaled lever produced a reinforcer (on average) every three minutes. The results showed no statistically significant differences between the groups under the VI 3 s schedule (Figures 1, 2 and 3; sessions 8-12).

The statistical analyses of the VI 180 s condition found no significant differences between the PCB 52 and controls. In contrast, animals exposed to PCB 153 and PCB 180 were significantly less active than the controls (Figure 2; sessions 20-33), and stimulus control was significantly better in the PCB 180 group compared to controls (Figure 1; sessions 20-33). Also, there was a tendency for the controls to collect more reinforcers than the PCB 153 group. However, this difference was small, not statistically significant, and the same significant effects were found when reanalyzing the data controlling for reinforcers collected.

Comparisons of the PCB groups showed that there were no significant differences between the PCB 153 and PCB 180 groups. However, both the PCB 180 and the PCB 153 groups were less active and had fewer responses with short IRTs (< 0.67 s) than the PCB 52 group (Figures 2, 3; sessions 20-33), and stimulus control was significantly better in the PCB 180 group compared to the PCB 52 group (Figure 1; sessions 20-33).

*Effects of exposure to PCB 52*. No behavioral changes were observed following exposure to PCB 52 in the present study. Boix et al. exposed female Wistar rats to PCBs at a dose of 1 mg/kg bw from GD 7 to PND 21 and tested the offspring when they were 3-4 months old [33]. Exposure to PCB 52 impaired motor coordination but did not affect Y maze performance. In two studies, Eriksson et al. reported learning deficits and behavioral changes in adult mice following exposure to a single dose of PCB 52 on PND 10 at a maximum dose of 4.1 mg/kg bw [27,52]. Kodavanti et al. exposed adult male Long-Evans rats to the PCB mixture Aroclor 1254 containing PCB 153, PCB 180 as well as PCB 52 [5]. On dissection, PCB 153 and PCB 180 were found in the brain, while PCB 52 could not be detected despite also having been present in the mixture. The PCB 52 congener is less chlorinated, and therefore, more soluble and less stable than PCB 153 and PCB 180, and may affect the brain differently than the two other congeners [4,32,33,53,54].

*Effects of exposure to PCB 153 and PCB 180*. When responding on the VI 180 s schedule, a marked significant decrease in lever-directed activity was observed in animals exposed to PCB 153 or PCB 180 compared to controls. However, although lever-directed activity (related to motivation and effects of reinforcers) was reduced in the exposed animals, it is unclear how general locomotor activity was affected in the exposed animals as this was not measured during the operant task. While visual inspection of Figure 3 reveals a reduced tendency for PCB 153 and PCB 180 rats to emit short IRTs, no significant differences between these groups and the control group were found. Only when the PCB 153 and PCB 180 groups were compared to the PCB 52 group did these comparisons reach statistical significance. For the stimulus control measure, only the PCB 180 group was significantly different from the control (and PCB 52) group.

In our experiment, stimulus control was inversely related to lever-directed activity. Theoretically, stimulus control is independent of rate of lever-pressing, and previous studies using an identical procedure have shown that these measures can be independently affected by experimental manipulations like exposure to drugs [46]. Still, it is possible that activity level is linked to exploration of other response alternatives, especially when reinforcement rate is low, and that the better stimulus control was secondary to hypoactivity produced by the PCB exposure. Further, the reduced number of responses with short IRTs found in the present study could be secondary to motor problems and hypoactivity produced by PCB exposure.

Studies of PCB 153 or PCB 180 have found impaired maze-learning in both male and female offspring of Wistar dams treated orally with PCB 153 from GD 7 to PND 21 at a dose of 1 mg/kg body weight per day, and in female offspring of Wistar dams treated with PCB 180 using the same dosing regimen [31,33,55]. Holene et al. mated female DA/OLA/HSD rats with Lewis rats and gavage-fed the dams PCB 153 every second day from PND 3 to 13 at a dose of 5 mg/kg [34,35]. The results showed that male, but not female, offspring became hyperactive, had more responses with short IRTs, and showed decreased stimulus control relative to controls in an operant test similar to the one used in the present study. Berger et al. found similar results in adult male Sprague-Dawley rats exposed during puberty to PCB contaminated fish or to contaminated diet containing low doses of the PCB mixture Aroclor 1248 (30 g daily portions of rodent diet supplemented with 1 ml corn oil containing 0.5 μg/g Aroclor 1248) [56]. These findings are opposite to the findings in the present study, and may be due to the different doses and dosing regimen used in the studies.

A range of conflicting results have been obtained in studies of PCB mixtures like Aroclor 1254 (containing both PCB 153 and PCB 180). Studies have reported changes in motor functions in young offspring of female Sprague-Dawley rats exposed to A1254 at a dose of 10 mg/kg/day from GD 11 to PND 21, and long-lasting hypoactivity in adult Long-Evans rats exposed to acute high doses (300 mg/kg and higher) or transitory hypoactivity after repeated exposure to doses of 30 mg/kg or higher [57,58]. Changes have also been observed in radial maze performance in adult male offspring of Long-Evans dams exposed to Aroclor 1254 at a dose of 6 mg/kg/day from gestational day 6 to PND 21, and spatial alternation deficits have been found in adult male and female offspring of Long-Evans dams exposed to 6 mg/kg from 28 days before mating to PND 16 [53,59]. Still, other studies have found no changes in activity or attention in adult offspring of Long-Evans dams exposed throughout gestation and nursing to Aroclor 1254 at doses of 1.0 or 6.0 mg/kg/day, or in spatial learning in young and adult offspring of Long-Evans dams exposed to 6 or 8 mg/kg/day from gestation to weaning [9,60-62].

PCB dose is probably one of several important factors determining the degree of cognitive and behavioral changes following exposure. Nishida et al., Eriksson et al., and Kodavanti et al. found a dose-dependent reduction in activity, whereas Holene et al. and Berger et al. found an increased activity level after exposure to different doses of PCB [5,27,34,56,58]. The combined findings suggest a curvilinear dose-response relationship or that the dose-response relationships are different for different brain regions, functions, and behaviors [25,27,63,64]. A range of other critical factors are likely to contribute to the varying results in studies examining effects of PCB exposure. The organism's age when exposed importantly influences the degree of neurological, cognitive, and behavioral changes. The nervous system seems to be more sensitive to toxins during development when there is a rapid growth and maturation of the brain [28]. Also, the age of the organism when tested and the measures used to assess the effects of exposure are important variables [31]. Findings show that the cognitive and behavioral changes following PCB exposure vary depending on the organism's age at the time of testing and on the measures used [27,31,33]. Additionally, effects of exposure to PCBs may be both sex- and species-specific [30,33-36,53]. In the present study, behavioral differences between groups were found only when reinforcers were delivered on the average every three minutes, and not during frequent reinforcement, consistent with previous results using the same procedure [39,56,65,66]. Thus, PCB exposure does not necessarily produce general behavioral problems. Exposed animals may perform normally under some test conditions (e.g. frequent reinforcement), but abnormally under others (infrequent reinforcement), adding to the argument that neurobehavioral effects of PCB exposure depend on a number of factors like procedures and measures used, age at exposure and at testing, and dose and type or combination of PCBs used.

*Limitations and future challenges*. The number of animals in the control group was smaller than originally planned. To assess the original control group, a second control group consisting of seven animals was tested. These animals were littermates of the animals in the original control group, one week older at the start of behavioral testing, and had not been given corn oil. These animals were not included in the main analyses because differences in age and corn-oil administration may have affected the behavioral measures. Therefore, the following analyses must be interpreted with caution. Statistical comparisons of the original and the second control groups showed no significant differences in measures of stimulus control, activity, or responses with short IRTs during VI 180 s. The extra controls were added to the original control group and all data were reanalyzed. The results for activity level and responses with short IRTs were in accordance with the main analyses, indicating that the findings were not produced by a small and non-representative control group. The analyses of stimulus control, however, did not reach the conventional level of significance (*p = 0.09*) suggesting that PCB exposure had little effect on stimulus control or that age differences in the added controls have affected the results.

Together with contradicting results from other studies, the present findings raise a call for systematic studies of dose-effects relations in PCB research. Studies need to include neurochemical as well as behavioral measures to uncover the underlying neurochemical changes. One contributing factor to the behavioral changes following PCB exposure may be alterations in dopamine function [2]. Hence, PCB exposure may play a role in the etiology of Attention Deficit/Hyperactivity Disorder and Parkinson's disease where dopamine disturbance is a likely cause [2,67-70]. Studies investigating a possible vulnerability to PCB exposure in these disorders using animal models should therefore be conducted.

## Conclusion

The present study found reduced activity in rats postnatally exposed to PCB 153 and PCB 180, while less robust result were obtained for stimulus control and responses with short IRTs. No effects of exposure to PCB 52 were found. The behavioral changes in the animals exposed to PCB 153 or PCB 180 were observed five weeks following the last exposure suggesting that exposure has long-lasting effects. Dose-response relations need to be established to determine whether this conclusion is valid also for other doses.

## Competing interests

The authors declare that they have no competing interests.

## Authors' contributions

EBJ participated in designing the study, and had the main responsibility for analyzing the data and drafting the manuscript. MK participated in data collection, and had with EBJ the main responsibility for the data analyses and drafting the manuscript. FF participated in designing the study, made the PCB-solutions used in the study, and helped drafting the manuscript. PLL participated in designing the study, had the main responsibility for animal breeding and welfare, administered the PCBs, and helped drafting the manuscript. SIV participated in designing the study and helped drafting the manuscript. GW was the main organizer of animal testing and data collection, performed preliminary data analyses, and helped drafting the manuscript. TS was the coordinator of the study, participated in the design and in drafting the manuscript. All authors read and approved the final manuscript.
